# Pilot Study to Demonstrate Improvement in Skin Tone and Texture by Treatment with a 1064 nm Q-Switched Neodymium-Doped Yttrium Aluminum Garnet Laser

**DOI:** 10.3390/jcm13051380

**Published:** 2024-02-28

**Authors:** Girish S. Munavalli, Hayley M. Leight-Dunn

**Affiliations:** 1 Department of Dermatology, Wake Forest University School of Medicine, 1918 Randolph Rd., Suite 550, Charlotte, NC 28207, USA; 2 Dermatology, Laser, and Vein Specialists of the Carolinas, 1918 Randolph Rd., Suite 550, Charlotte, NC 28207, USA; hleightdunn@carolinaskin.com

**Keywords:** dark skin types, melanin, Nd:YAG laser, pigmentation, laser toning, facial aging

## Abstract

Background: The 1064 nm Q-switched neodymium-doped yttrium aluminum garnet (QS Nd:YAG) laser was developed to treat unwanted pigmentation in the skin such as lentigines caused by photoaging, and tattoos from dye/ink insertion. This laser has also been used for non-ablative epidermal rejuvenation (skin toning). Objective: To evaluate changes in skin tone, skin texture and overall improvement after a series of treatments with the QS Nd:YAG laser. Methods: Participants received seven full-face treatments with M22 or Stellar M22, a 1064 nm QS Nd:YAG laser, at 2-week intervals. The investigators and participants evaluated the improvement in skin tone and texture at 1, 3 and 6 months after the last treatment. Patient satisfaction, patient discomfort, downtime and adverse events were recorded. Histological changes in the treated area were also evaluated. Results: Thirteen women with a median age of 45 years (range, 34–61 years) were included in the study. The majority of the participants (53.9%) had skin type VI. One month after the last treatment session, 38% of participants reported good to very good improvement. This value increased to 100% participant improvement at both the 3-month and 6-month follow-up visits. The reduction in melanin index and the histological analysis demonstrated that the laser procedure contributed to a reduction in epidermal melanin content. Treatments were not associated with high levels of pain or discomfort. The most common immediate post-treatment response was erythema and edema. Most participants were satisfied with the resulting treatment outcome. Conclusion: Skin treatment with the 1064 nm QS Nd:YAG laser module on the M22 and Stellar M22 devices, using a large spot size, low fluence, moderately high repetition rate, improves skin tone and texture in patients with skin types II–VI.

## 1. Introduction

The Q-switched neodymium-doped yttrium aluminum garnet (QS Nd:YAG) laser was developed to treat skin pigments such as those present in tattoos and lentigines. Despite the lower absorption of the 1064 nm wavelength by melanin compared to the red and green laser wavelengths, the 1064 nm energy penetrates more deeply into skin. This renders the 1064 nm wavelength an advantage over the other lasers in the treatment of patients with skin of color [1].

Nanosecond pulses emitted by the QS Nd:YAG are absorbed by melanosomes or tattoo particles and converted to heat, ultimately leading to fragmentation of the pigment particles. The rapid and selective nature of this process prevents collateral damage to adjacent normal tissue. However, the subthreshold injury to the surrounding dermis stimulates the formation of collagen. The pigments become lighter through successive treatments in which areas of epidermal and dermal tissue are destroyed, subsequently eliminating them from the skin surface [2,3,4]. This process results in brighter and tighter skin [5], leading to improved skin tone [6,7]. 

In 2012, Kauvar [4] reported promising results in the treatment of melasma with a low-fluence QS Nd:YAG laser. The laser selectively targets dermal melanosomes without producing inflammation or epidermal injury in all skin phototypes. That same year, Arora et al. [8] reported that treatment with low-fluence QS Nd:YAG laser, or “laser toning”, had become popular not only for the treatment of melasma, but also for skin rejuvenation in Asian patients. In laser toning, treatment parameters include the large spot size (6–8 mm), low fluence (1.6–3.5 J/cm^2^), and multiple passes performed every 1 to 2 weeks for several weeks. As a result of these findings, laser toning procedures became a common request in patients with pigmented skin.

Research on the molecular mechanism of QS Nd:YAG irradiation in cells and animal models has shown a variety of integrated molecular pathways. QS Nd:YAG irradiation can downregulate the expression of matrix metalloproteinases (MMP) 1 and 2, which are increased in damaged skin, and upregulate the expression of Collagen Ⅰ and Collagen Ⅳ through the miR-633a transforming growth factor-β/Smad3/p38MAPK axis. Moreover, other clinical research has shown that QS Nd:YAG irradiation inhibits trans-epidermal water loss and promotes moisture content, elasticity, hydroxyproline content and superoxide dismutase [9,10,11,12]. In addition, the QS Nd:YAG laser was shown to effect the skin barrier function, increasing the expression of aquaporins, filaggrin, transglutaminase and heat-shock protein 70 [13].

The purpose of this study was to evaluate the safety profile and changes in skin tone and texture following a series of treatments for diffuse facial hyperpigmentation with a 1064 nm QS Nd:YAG laser. Such causes of hyperpigmentation can be due to post-inflammatory changes from chronic skin irritation, contact dermatitis, eczematous dermatitis, or more regionally on the face in the case of post-acne hyperpigmentation. While this study was open to participants with all skin types, particular attention was given to highlight the results for participants with skin type VI given the complexity of using a laser and the higher risk of laser complications in this patient subset.

## 2. Methods

### 2.1. Population

This was an open-label study conducted at 2 sites. Healthy patients aged 30–65 years of age with Fitzpatrick–Goldman skin type I-VI requesting laser treatment of pigmentation or textural improvement were eligible to participate in the study. Woman of child-bearing age had to use a reliable method of birth control at least 3 months prior to enrollment and throughout the study. Patients were excluded if they had symptoms of hormonal disorders (e.g., melasma or chloasma), pigmented melanocytic lesions in the treatment area or other skin pigmentation disorder, dermal or epidermal damage or disorder (e.g., vascular or textural lesions), excessive erythema, keloids, hypertrophic scar or prescar formation, an open wound or abrasion or a history of poor wound healing, concurrent inflammatory skin conditions, a history of post-inflammatory hyperpigmentation, skin hypopigmentation, active herpes simplex infection, bleeding disorders or any other undesired response deemed by the investigator as grounds for exclusion. Participants with a history of rejuvenation treatments in the area to be treated (up to 12 months before the study), allergies to anesthetics, women who intended to become pregnant during the study period and women who were pregnant or breastfeeding were also excluded.

Thirteen women with a median age of 45 years (range, 34–61 years) were included in the study. Two women (15.4%) had skin type II, three (23.1%) had skin type III, one (7.7%) had skin type IV and seven women (53.9%) had skin type VI. No women with skin type I or V participated in this study. All patients completed the 1-month follow-up visit; 8 participants completed the 3-month follow-up visit and 5 participants completed the 6-month follow-up visit.

The study was approved by the institutional review board and all patients provided signed informed consent to participate.

### 2.2. Treatment

Each participant received 7 full-face treatments with the 1064 nm QS Nd:YAG laser module using Lumenis QS Nd:YAG laser module (Lumenis Be Ltd., Yokneam, Israel) at 2-week intervals. Optimal settings for the laser were determined by pretreatment test spots in the treatment area.

Seven participants (all with skin type VI) were treated with a single pass over the designated area using a 6 mm spot size, 1.6 J/cm^2^ fluence, and 2–4 Hz repetition rate. The rest of the participants (with skin types II-IV) were treated with an 8 mm spot size, 0.9 J/cm^2^ fluence, 3–5 Hz repetition rate and multiple passes (3–5 passes). Repetition rates were initially low and increased with subsequent treatments. Topical numbing with lidocaine/tetracaine 6%/6% was applied 30 min before treatment for some patients (of skin type VI) based on their response to the first treatment. The endpoint for treatment was mild erythema. If urtication or immediate dermal swelling was seen during the treatment, the energy was reduced by 10–20%.

### 2.3. Assessments of Outcomes

Digital photographs were taken with a high-quality camera (Canon EOS Rebel T3i, ISO-speed of 400 preset using the FotoFinder R3 system software) at baseline, and at the 1-, 3- and 6-month follow up visits.

Outcome assessments included the investigator’s subjective evaluation of improvement in skin texture and skin tone as well as overall improvement at 1, 3 and 6 months after the last treatment session. Improvements in skin tone and texture and overall improvement were categorized as none (0%), slight (1–25%), moderate (26–50%), good (51–75%) and very good (76–100%). The participants also self-evaluated their skin improvement following the treatment and their satisfaction with the treatment series.

The absolute melanin index, an objective measure of skin tone, was measured with a Mexameter^®^ (Courage-Khazaka Electronic GmbH, Köln, Germany) on the right and left sides of the face at multiple points within the treatment area. The Mexameter measures absorbed and reflected light at green and red wavelengths for hemoglobin and at the red and near-infrared wavelengths for melanin. The instrument computes the melanin index from the intensity of the absorbed and reflected light at, respectively, 660 and 880 nm. The Mexameter can detect small differences in skin color among all skin types [14]. Melanin indices were measured in two anatomical areas at baseline; treatments 3, 5, and 7; and at 1, 3 and 6 months after the last treatment session. Differences at each time point from baseline were calculated and were an indicator of response to treatment, as suggested by Sethuraman et al. [15].

Skin response to laser treatment (erythema, edema, hives) was graded using a 4-point scale (1 = trace; 2 = moderate; 3 = marked; 4 = severe) within 20–30 min of completion of each treatment.

To evaluate pain and discomfort associated with the laser treatments, the participants were asked to rate their general pain and comfort level immediately after each treatment using a visual analogue scale (VAS) ranging from 0 (no pain) to 10 (intolerable pain).

Participant-reported downtime was defined as the period of time following the procedure during which the patient had edema and erythema and was uncomfortable and unwilling or unable to go out in public. Complications and adverse events were also monitored and recorded.

### 2.4. Histological Analyses

Three patients consented to excision of two 3 mm sized pre-auricular punch biopsy samples. For the control site, a small preauricular area was marked prior to each treatment and the laser treatment was avoided in this small area. One sample was taken from a laser-treated area and the other from an untreated (control) area in the same participant at 1 month and 6 months after the last treatment session. Samples were excised following injection of 1–2% lidocaine with epinephrine. The excised samples were evaluated histologically for dermal modifications that occurred. Each sample was fixed in 4% formaldehyde, processed, and embedded in paraffin before being cut into 5 µm sections. The sections were then stained with Hematoxylin and Eosin (H&E) and melanin-specific Fontana Masson’s (FM) stain. Histological sections were viewed under Leica DMRM light microscopy attached to a Nikon DS-Fi2 CCD camera and analyzed by NIS-Elements BR software (Nikon, Tokyo, Japan).

## 3. Results

### 3.1. Skin Improvement

**Figure 1:**
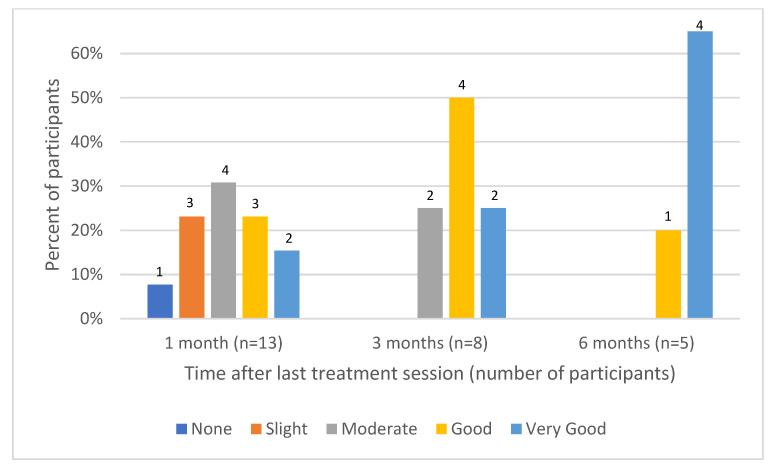
Participant-evaluated skin improvement.

**Figure 2:**
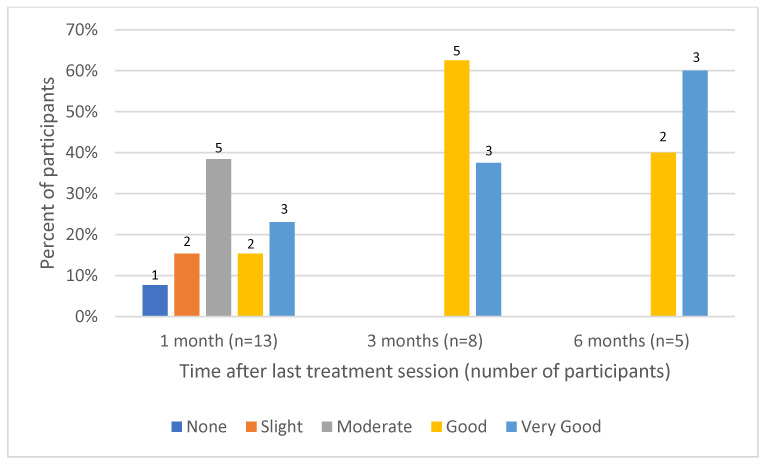
Participant satisfaction with the treatment.

Data regarding participant self-evaluation at 1, 3 and 6 months after the last treatment session were available for 13, 8 and 5 participants. One month after the last treatment session, 5 of 13 participants (38%) reported good to very good improvement, which increased to 100% at 3 and 6 months after the last treatment session (Figure 1). The percentage of patients reporting good to very good satisfaction with the treatment also increased from 38% at 1 month of follow-up to 100% satisfaction at 3 and 6 months (Figure 2).

**Figure 3:**
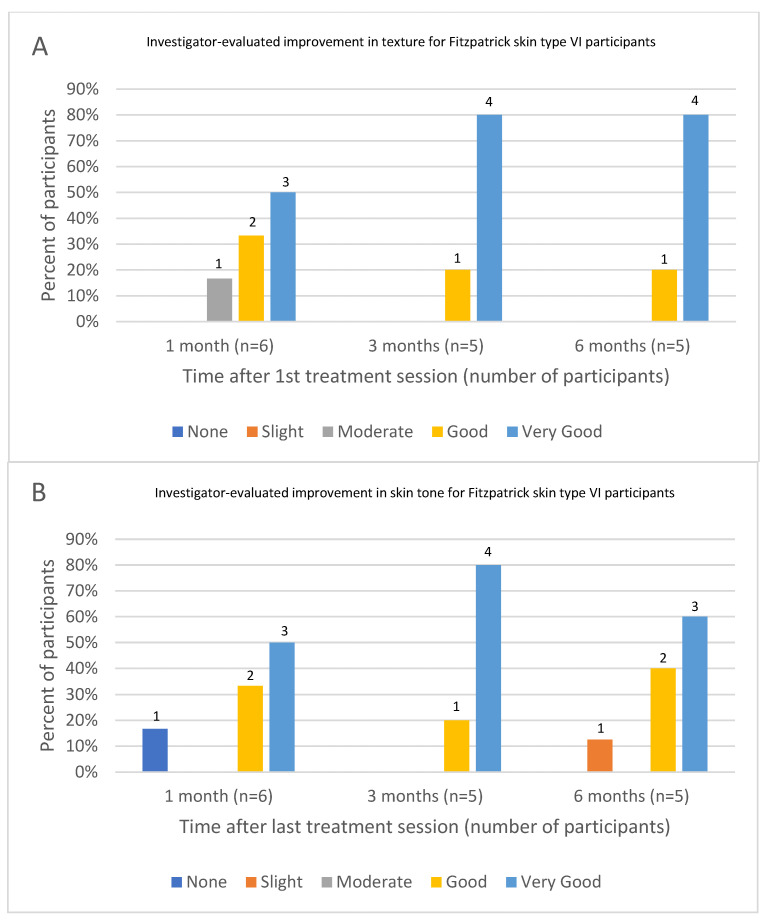

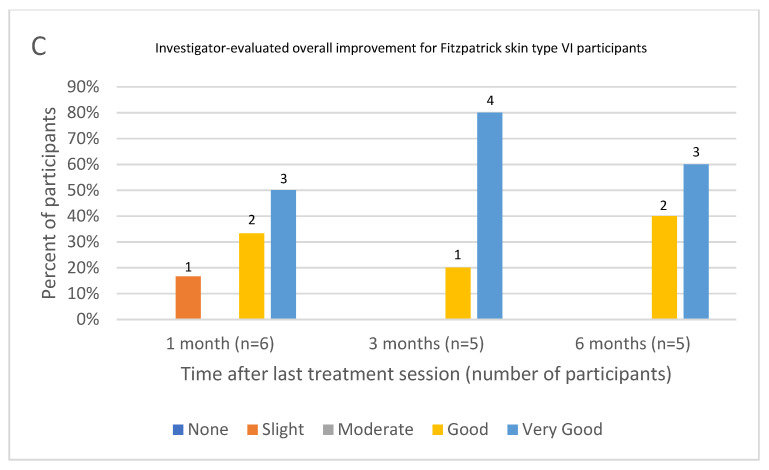
Investigator-evaluated skin improvement for Fitzpatrick skin type VI participants. (**A**) Investigator-evaluated improvement in texture. (**B**) Investigator-evaluated improvement in skin tone. (**C**) Investigator-evaluated overall improvement.

Investigator assessments of changes in skin tone and texture for Fitzpatrick skin type VI participants at 1-, 3- and 6-months follow-up were available for six, five and five participants, respectively. Five participants demonstrated good to very good improvement in skin tone, texture and overall improvement, at all follow-up timepoints (Figure 3A–C). At the one-month follow-up, one participant demonstrated only moderate improvement in skin texture and no improvement in skin tone. This participant withdrew her consent for participation in the study and was not evaluated in subsequent visits.

**Figure 4:**
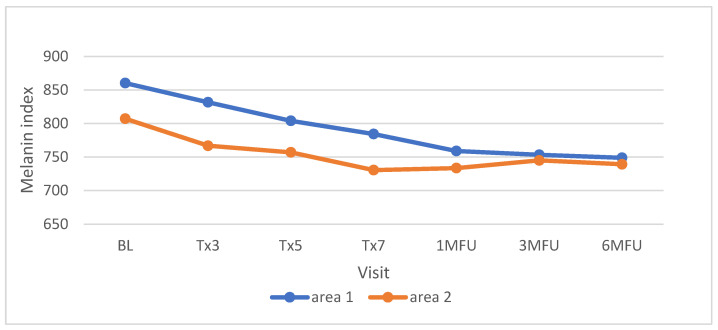
Mean change in melanin index during the study (area 1 = left side of the face, area 2 = right side of the face).

The absolute melanin index, which reflects the brightness of the skin, was measured in 6 participants with skin type VI. The mean melanin index at baseline was 860.3 and 807.2 on the left and right sides of the face, respectively. At the final visit (6 months follow up), the mean levels decreased to 749 and 739.2, respectively (Figure 4).

### 3.2. Histology

**Figure 5:**
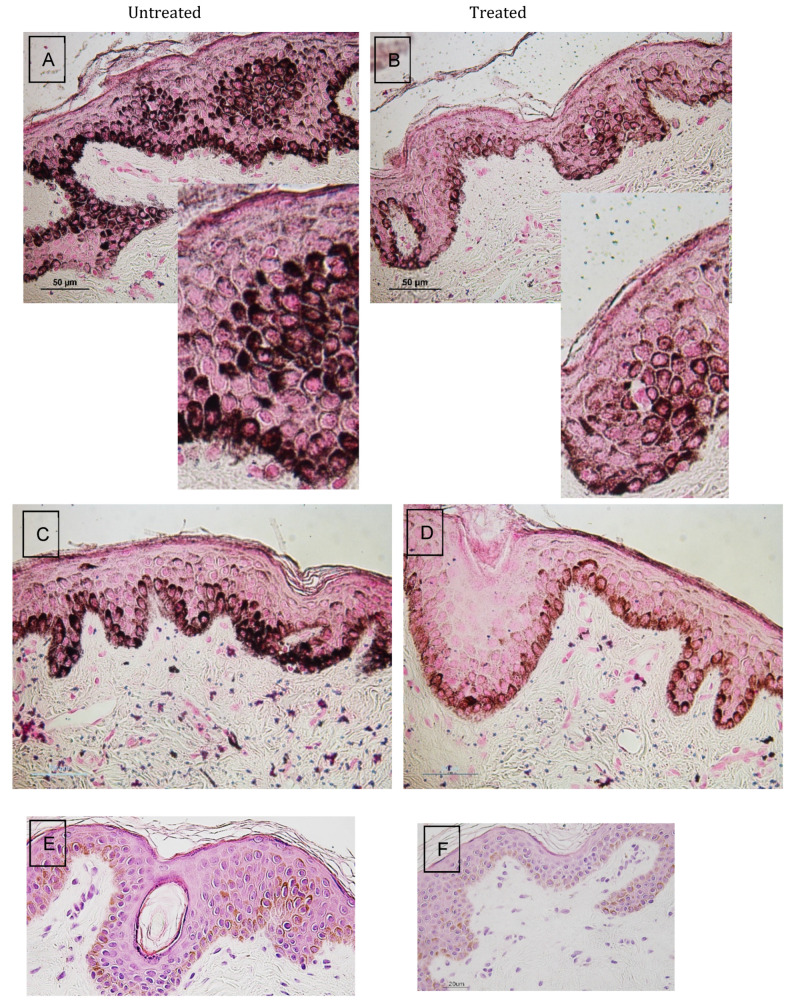

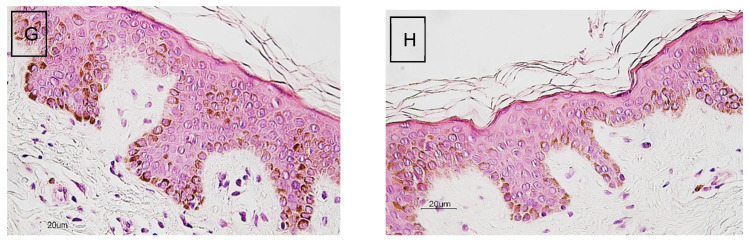
Representative histological section of pre-auricular punch biopsy samples stained with melanin-specific Fontana Masson’s stain. (**A**,**C**) Untreated samples 1 and 6 months, respectively, after the last treatment session; (**B**,**D**) laser-treated samples 1 and 6 months, respectively, after the last treatment session; (**E**,**G**) Hematoxylin and Eosin stain of untreated samples 1 and 3 months, respectively, after the last treatment session; (**F**,**H**) laser-treated samples 1 and 3 months, respectively, after the last treatment session.

Stained melanin (using Fontana Mason’s dye) was seen abundantly in the basal layer of the epidermis of untreated samples (Figure 5A,C), as well as in the upper layers of epidermis, whereas less intense staining was seen in the same layers of the laser-treated samples (Figure 5B,D). Similar observations were noted following staining with H&E (Figure 5E–H).

### 3.3. Safety

Safety was evaluated in all patients. During most treatments, they reported feeling slight to moderate pain, with an average discomfort level of 5 for all the treatments (range, 2–8.3). In some cases, the assessed pain levels were lower in subsequent treatment sessions in comparison to the first treatment.

Immediately after each treatment, the response of the treated skin was observed and noted for each patient. The intensity of edema was mostly trace (57% of responses) or moderate (6% of responses). As the treatments progressed, it was noted that the erythema response in the skin did not change and remained trace; however, in some participants, the edema response decreased to trace. Two patients developed urticaria at subsequent visits. No patients experienced severe responses at any treatment visits. Edema and swelling lasted less than a day in most patients (median, 0.85 and 0.95, respectively).

Ten adverse events were reported by five participants with dark skin (skin type VI). Among these events, three were anticipated laser-treatment-related adverse effects. Treatment-related adverse events included peeling in one participant, itching in another and prolonged edema lasting for 6 days in a third participant. Three patients experienced treatment-related hypopigmentation, with one patient experiencing it for 7 days and the other two for 6 months. Additionally, two participants had herpes simplex and one participant developed a hypertrophic scar at the bilateral biopsy site, which was considered to be unrelated to the treatment.

## 4. Discussion

**Figure 6:**
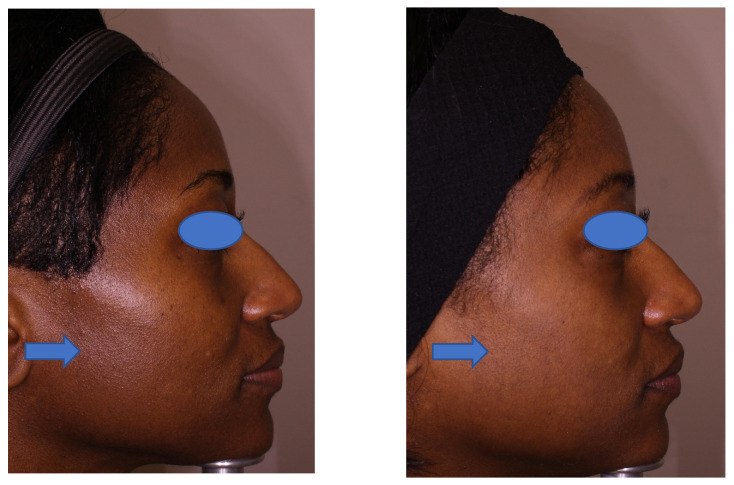
A 46-year-old, skin type VI, female at baseline (**left** photo) and at the 3-month follow-up visit (**right** photo). This patient achieved very good improvement in skin tone, skin texture and overall improvement 3 months after a series of 7 treatments with the 1064 nm Q-switched Nd:YAG laser.

**Figure 7:**
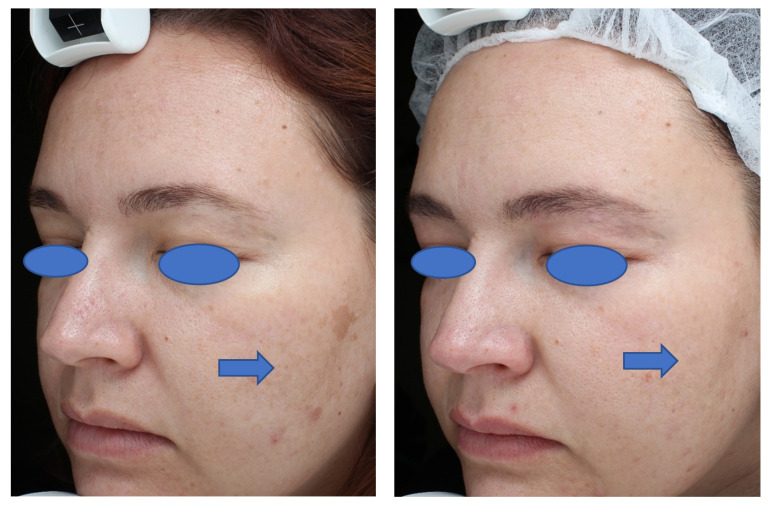
A 37-year-old, skin type III, female at baseline (**left** photo) and at the 3-month follow-up visit (**right** photo). This patient achieved very good improvement in skin tone, skin texture and overall improvement 3 months after a series of 7 treatments with the 1064 nm Q-switched Nd:YAG laser.

The study results indicate that a series of seven treatments using the 1064 nm QS Nd:YAG laser improved skin tone and texture, particularly in patients with skin type VI. Moreover, use of this laser in broader skin-type categories II through VI was also associated with a high level of participant-evaluated skin improvement and participant satisfaction. The reduction in melanin index and histological data demonstrate that the laser procedure contributed to a reduction in epidermal melanin content as well. The improvement is apparent in the pre- and post-treatment photographs, in which patients present a brighter and less “oily” skin appearance at the follow-up visits (Figure 6 and Figure 7). Participants were satisfied with the treatment outcomes up to 6 months after the last treatment session.

Treatments of skin aging in patients with dark skin, in particular skin type V and VI, present a unique challenge to practitioners. In Caucasian skin, photoaging, rhytids and skin laxity prevail. These different concerns can often be treated with a myriad of different lasers and procedures. In darker skin types, aging often manifests itself with changes in the skin texture and pigmentary changes, providing an excellent opportunity for laser skin rejuvenation with the QS Nd:YAG laser [16,17]. Darker skin types have a propensity for laser complications, such as hyperpigmentation, hypopigmentation and scarring. Therefore, it is critical to determine safe and effective treatments for patients with darker skin types. Laser toning has thus become popular for this subset of patients. Laser toning for skin types III to IV is typically performed with low fluence (1.6–3.5 J/cm^2^), large spot size (6–8 mm), multiple passes, and 1- to 2-week treatment intervals using mild erythema as the primary end point [18]. Since it is difficult to note any erythema as an end point for skin types V to VI, treatment of individuals with skin type VI was limited to a single pass over the target area. Following our study, we recommend using the biggest spot size (8) when treating skin type 6.

Our results align with those reported by others. Lee et al. [7] treated individuals with skin types III and IV with the QS Nd:YAG laser four times at 4-week intervals and demonstrated improvement in rough skin surface texture, skin tone, pore size and sebum production. Won et al. [19] treated 13 Korean women with QS Nd:YAG laser at 1-week intervals and concluded that in Korean women (Skin type IV) with melasma, good to excellent improvement could be achieved by laser toning with 6 mm spot size and 2.5 J/cm^2^ for melasma and, for darker spots, 4 mm spot size and 4 to 5 J/cm^2^ with two passes and seven treatment sessions. Agarwal et al. [20] treated 252 patients with skin types III-VI with QS Nd:YAG laser for skin rejuvenation. The patients underwent biweekly sessions for a total of six sessions. After the first session, immediate improvements in skin texture and tone were observed that increased over three sessions and then stabilized. No downtime was reported [20].

In another study conducted by Luebberding and Alexiades-Armenakas, treatment of seven women with a fractional, non-ablative QS Nd:YAG laser (spot size 5 mm, 400–1200 mJ/pulse, repetition rate 4 Hz, 8–12 passes) every 2–4 weeks for a total of three sessions significantly improved superficial rhytides and was deemed to be particularly suitable for the treatment of sensitive areas, such as the periorbital region, lips, neck and chest [21]. Similarly, three treatment sessions at 2-week intervals with a fractional QS Nd:YAG laser treatment (2400 mJ/pulse, repetition rate 3 Hz and a fixed pulse time of 20 ns, two passes per session) of 16 women aged 36–54 years with skin phototypes II, III, and IV and mild to mild–moderate skin aging of the face and neck also showed a mean reduction of 30–40% in signs of skin aging evaluated by the Global Esthetic Improvement Scale [22]. In a split-face study, repeated treatments with QS Nd:YAG laser (spot diameter 3–4 mm, fluence 7–9 J/cm^2^, 10 Hz frequency) effectively removed the pigment in 28/29 individuals with Nevus of Ota and Fitzpatrick skin types III or IV and improved facial appearance by 10–60%. Statistically significant improvement in wrinkles and skin texture was observed after an average of eight treatments; the skin had less pigmentation, a more delicate texture, less drooping of the outer canthus and a significantly lighter nasolabial sulcus, compared with the untreated side. The degree of skin rejuvenation was positively correlated with the number of treatment sessions [23].

We examined the histological changes in the skin following laser treatment using staining techniques. Melanin is synthesized by melanocytes aligned along the proliferative section of the basal layer of the epidermis. Melanin undergoes an intercellular transfer from the melanocyte to the keratinocytes, located in the upper layers of epidermis (stratum spinosum). Stained melanin was seen abundantly in the basal and upper layers of the epidermis of untreated samples, whereas a less intense stain appeared in the same layers of the treated samples.

In this study, treatments were not associated with high levels of pain or discomfort. In some cases, the assessed pain levels were lower in subsequent treatment sessions. This may be a result of the patient becoming accustomed to the treatment sensations or due to decreased pigment in the treatment area.

The most common immediate post-treatment response was erythema and edema, persisting for one day in most participants. One participant had more prolonged edema for 6 days, but in the author’s opinion and expertise this is not a common post-treatment response seen with the 1064 nm QS Nd:YAG laser treatments. Laser toning with the low-fluence 1064 nm QS Nd:YAG laser by other groups [18,24] has previously been associated with complications in Asian patients. Chan et al. [18] reported facial depigmentation in patients who received 6 to 50 treatments given at intervals ranging from daily to monthly. Treatments were stopped when mottled facial depigmentation developed. Wong et al. [24] reported the development of guttate hypopigmentation in three patients, one of whom received 40–50 laser treatments over 6 months at intervals ranging from several times daily to weekly. The authors concluded that laser toning treatment should be given no more frequently than once every fortnight and the total number of treatments should be limited to avoid the development of hypopigmented macules. The incidence of PIH following 1064 nm QS Nd:YAG laser treatment ranges from 18% to 73% in Asian participants [25,26,27]. In a meta-analysis of 43 studies that aimed to determine the types and rates of adverse events in non-ablative laser and energy-based therapies among patients with Fitzpatrick skin types IV to VI, the most common adverse events were PIH (8.1%) and prolonged erythema (0.6%) [28]. PIH was not observed in the current study. In the author’s experience and opinion, the risk of PIH is greater with shorter treatment intervals as opposed to the total number of treatments overall. Other potential complications of laser toning with the 1064 nm QS Nd:YAG laser, particularly in individuals with darker skin types, include mottled hypopigmentation, herpes simplex reactivation, acneiform eruption, physical urticaria, minute petechiae, whitening of fine facial hair and leukoderma [25,29,30,31]. Three of the patients in this study developed treatment-related hypopigmentation that resolved in all cases. The cases of herpes simplex activation during the course of the study were deemed unrelated to the treatments. No other unexpected adverse events occurred.

This study has multiple limitations and is primarily limited by a small sample size and lack of follow-up from some of the participants. Moreover, participants who did not see improvements at the one-month follow-up withdrew their consent to participation in the study, limiting the evaluation of changes to the skin in further follow-up timepoints. This potentially caused bias in our results and the conclusions may have been less positive if those patients had returned for follow-up visits. As with all pigmentary skin conditions, changes in skin color can also be seen with seasonal variations, which can influence laser study results.

In conclusion, despite these limitations, it is evident that skin treatment with the 1064 nm QS laser improves skin tone and texture in Fitzpatrick skin type VI participants and has high patient satisfaction and participant-evaluated skin improvement for a wide range of skin types (II–VI). Histologic improvements in laser-treated skin also provide an objective assessment to an often-subjective analysis of skin pigmentation. The 1064 nm QS Nd:YAG laser is well tolerated by participants with limited and well documented adverse reactions. Further studies are warranted to further clarify the efficacy and safety of the 1064 nm QS Nd:YAG laser in all skin types.

## Data Availability

There were no publicly archived datasets made available through this study.
